# Posterior column osteotomy plus unilateral cage strutting for correction of lumbosacral fractional curve in degenerative lumbar scoliosis

**DOI:** 10.1186/s13018-020-02011-y

**Published:** 2020-10-20

**Authors:** Hui Wang, Longjie Wang, Zhuoran Sun, Shuai Jiang, Weishi Li

**Affiliations:** 1 Orthopaedic Department, Peking University Third Hospital, No 49. North Garden Street, HaiDian District, Beijing, 100191 China; 2 Beijing Key Laboratory of Spinal Disease Research, Beijing, China; 3 Engineering Research Center of Bone and Joint Precision Medicine, Ministry of Education, Beijing, China

**Keywords:** Posterior column osteotomy, Unilateral cage strutting, Lumbosacral fractional curve, Degenerative lumbar scoliosis

## Abstract

**Background:**

Inadequate release of the posterior spinal bone elements may hinder the correction of the lumbosacral fractional curve in degenerative lumbar scoliosis, since the lumbosacral junction tends to be particularly rigid and may already be fused into an abnormal position. The purpose of this study was to evaluate the surgical outcome and complications of posterior column osteotomy plus unilateral cage strutting technique on lumbosacral concavity for correction of fractional curve in degenerative lumbar scoliosis patients.

**Methods:**

Thirty-two degenerative lumbar scoliosis patients with lumbosacral fractional curve more than 15° that were surgically treated by posterior column osteotomy plus unilateral cage strutting technique were retrospectively reviewed. The patients’ medical records were reviewed to identify demographic and surgical data, including age, sex, body mass index, back pain, leg pain, Oswestry Disability Index, operation time, blood loss, and instrumentation levels. Radiological data including coronal balance distance, Cobb angle, lumbosacral coronal angle, sagittal vertical axis, lumbar lordosis, and lumbosacral lordotic angle were evaluated before and after surgery. Cage subsidence and bone fusion were evaluated at 2-year follow-up.

**Results:**

All patients underwent the operation successfully; lumbosacral coronal angle changed from preoperative 20.1 ± 5.3° to postoperative 5.8 ± 5.7°, with mean correction of 14.3 ± 4.4°, and the correction was maintained at 2-year follow-up. Cobb’s angle and coronal balance distance decreased from preoperative to postoperative; the correction was maintained at 2-year follow-up. Sagittal vertical axis decreased, and lumbar lordosis increased from preoperative to postoperative; the correction was also maintained at 2-year follow-up. Lumbosacral lordotic angle presented no change from preoperative to postoperative and from postoperative to 2-year follow-up. Postoperatively, there were 8 patients with lumbosacral coronal angle more than 10°, they got the similar lumbosacral coronal angle correction, but presented larger preoperative Cobb and lumbosacral coronal angle than the other 24 patients. No cage subsidence was detected; all patients achieved intervertebral bone fusion and inter-transverse bone graft fusion at the lumbosacral region at 2-year follow-up.

**Conclusion:**

Posterior column osteotomy plus unilateral cage strutting technique on the lumbosacral concavity facilitate effective correction of the fractional curve in degenerative lumbar scoliosis patients through complete release of dural sac as well as the asymmetrical intervertebral reconstruction by cage.

## Background

Degenerative lumbar scoliosis (DLS) is a three-dimensional disorder that mostly affects the skeletally mature patients; the typical major curve lies in the mid-lumbar spine; it is always compensated by a distal curve as the body attempts to maintain coronal balance [1, 2]. Compensatory curve at the lumbosacral region below the major curve is named the fractional curve; the nerve roots on the concave side of the fractional curve are the most frequent radicular pain generators in DLS patients [3–5]. The neurologic symptoms derived from the nerve root compression is strongly correlated with the decision to pursue surgical treatment; spine surgeons must include in surgical planning the symptomatic radicular levels that are related to the fractional curve instead of the major curve [6]. It has been recommended that all fractional curves greater than 15° must be corrected and included within the instrumentation [7]. If a fractional curve is not addressed properly, the patient is very unlikely to obtain coronal realignment or may experience aggravation of the coronal imbalance; there is a growing body of literature correlating coronal imbalance with a negative effect on patient outcomes [8–11].

Various techniques to correct fractional curve have been described in the literature, including anterior lumbar interbody fusion (ALIF), oblique lumbar interbody fusion (OLIF), posterior or transforaminal lumbar interbody fusion (PLIF or TLIF) [4, 12]. ALIF could achieve better fractional curve improvement but is associated with potential risk of intra-abdominal vascular and visceral injury [13]. OLIF has been proved to correct fractional curve effectively through a minimally invasive incision; it still shares the risk of vascular injury [14]. The posterior insertion of cage into the concavity of the curve through PLIF or TLIF can restore the disc height and achieve lumbosacral curve correction [15]. A laminectomy would be performed medial to the facet in PLIF technique, while bilateral facet joints are preserved. In TLIF technique, the spinal canal is entered via a unilateral laminectomy and inferior facetectomy; the contra-lateral laminae and facet joint are preserved. Inadequate release of the posterior spinal bone elements in PLIF or TLIF technique may hinder the correction of the lumbosacral curve because the lumbosacral junction tends to be particularly rigid and may already be fused into an abnormal position.

Posterior column osteotomy (PCO) is accomplished by resecting bone and ligaments between the posterior columns of adjacent spinal segments, leaving only the mobile disc anteriorly through which correction is obtained [16–21]. Principal indications for PCO include reducing kyphosis or increasing lordosis on sagittal plane or increasing the flexibility of a stiff scoliosis on the coronal plane to enable correction [22]. It is reasonable to postulate that posterior insertion of cages into the concavity of the curve followed PCO could facilitate more fractional curve correction than PLIF or TLIF, since complete release of the posterior spinal column can be achieved through PCO. However, little evidence is specifically regarding the effect of PCO plus unilateral cage strutting (UCS) in the correction of lumbosacral fractional curve. The purpose of this study is to evaluate the surgical outcome and complications of PCO plus UCS on the lumbosacral concavity for the treatment of fractional curve in DLS.

## Materials and methods

### Patients

This was a retrospective study and was approved by the Institutional Review Board of our hospital before data collection and analysis. The inclusion criteria are as follows: (1) degenerative lumbar scoliosis with lumbosacral fractional curve more than 15°, which needs correction; (2) the lumbosacral fractional curve was surgically treated by PCO plus UCS technique; (3) minimum follow-up of 2 years; (4) full-spine posterior-anterior (P/A) and lateral X-ray at pre-op and post-operative. The exclusion criteria are (1) previous surgery for degenerative lumbar disease, (2) underlying diseases that seriously affected the surgical outcomes, and (3) the anatomical identification was difficult to recognize for radiological measurement. From Jan 2014 to Aug 2017, 237 DLS patients were surgically treated in our hospital and were retrospectively reviewed; 32 patients whom met both the inclusion and exclusion criteria were enrolled into this study; the main complaints were pain, walking difficulty, and deformity.

### Data collection

The patients’ medical records were reviewed to identify demographic and surgical data, including age at surgery, sex, body mass index (BMI), back pain (visual analog scale, VAS-back), leg pain (VAS-leg), Oswestry Disability Index (ODI), operation time, blood loss, instrumentation levels, and complications.

**Fig. 1:**
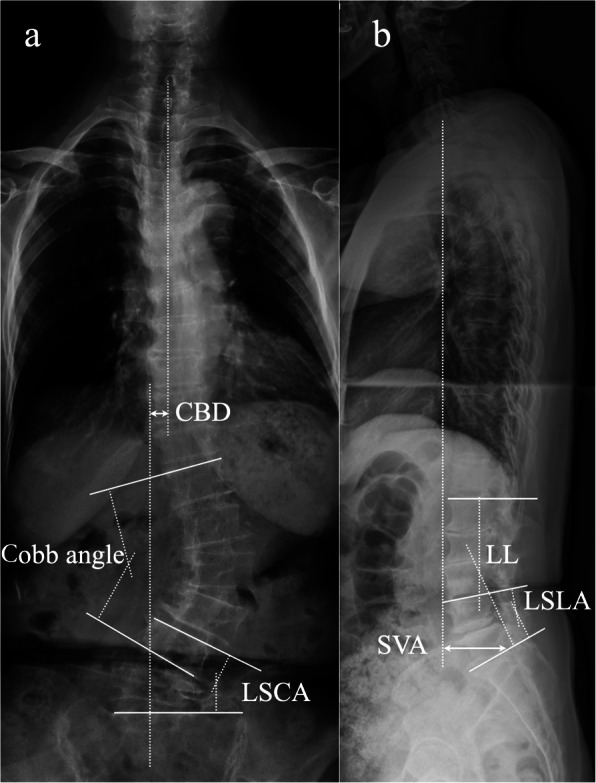
Schematic diagram of measured coronal and sagittal spinal parameters, including coronal balance distance, Cobb’s angle, lumbosacral coronal angle (**a**), sagittal vertical axis, lumbar lordosis, lumbosacral lordotic angle (**b**)

Standing P/A and lateral radiography was used to evaluate the spine curvature and alignment before surgery, immediately after surgery, at 3 months, and 2 years of follow-up. Coronal balance distance (CBD) was measured as the distance between C7 plumb line and center sacral vertical line (Fig. 1a). Type of coronal imbalance was evaluated in all the patients: type A, coronal balance distance (CBD) < 3 cm; type B, CBD > 3 cm and C7PL shifts to the concave side of the curve; Ttype C, CBD > 3 cm and C7PL shifts to the convex side of the curve [23].

Cobb’s angle was measured using lines projected from the upper border of the most tilted vertebra above and lower border of the most tilted vertebra within the main curve. Lumbosacral coronal angle (LSCA) was measured using lines projected from the upper border of L4 and upper border of S1 on P/A X-ray (Fig. 1a).

Sagittal vertical axis (SVA) was the distance from C7 plumb line to the perpendicular line drawn from superior posterior endplate of S1. Lumbar lordosis (LL) was measured from upper endplate of L1 to upper endplate of S1. Lumbosacral lordotic angle (LSLA) was measured using lines projected from the upper border of L4 and upper border of S1 on lateral X-ray (Fig. 1b).

**Fig. 2:**
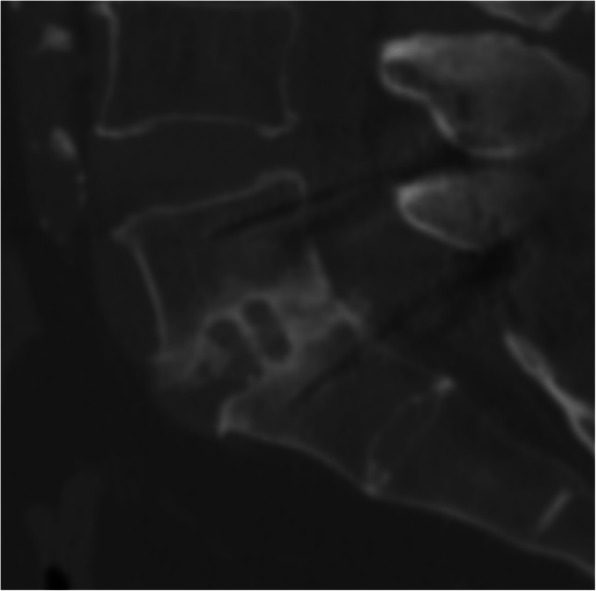
Cage subsidence is measured as 2 mm of cage settlement into the vertebral body

Cage subsidence was assessed utilizing lumbar spine CT taken at 2-year postoperatively; it was defined as 2 mm of cage settlement into the vertebral body (Fig. 2). Assessment of radiological bone fusion at follow-up was based on the presence of trabecular bone bridging at the osteotomy site according to Bridwell et al. [24].

### Surgical procedure

Under general anesthesia, all patients were placed in a prone position on a radiolucent table. A posterior midline incision was made for conventional exposure. Pedicle screws were inserted according to the surgical planning. PCO plus UCS was first performed within the lumbosacral region: The posterior elements that include the spinous process, bilateral laminae, bilateral pars interarticularis, bilateral inferior facet joints, and the medial and superior aspects of the subjacent superior facet joints were removed. The intervertebral foramen was explored; both the exiting nerve roots and the walking nerve roots were exposed and protected. An incision was made through the anulus; the degenerated nucleus pulposus and the cartilaginous endplate were removed by osteotome and rongeur. This procedure was performed bilaterally. On the concave side, the smallest reamer was horizontally inserted into the narrowed disc space and rotated 90°, thereby restoring the disc height and reducing the compensatory scoliosis. The maneuver was repeated using a larger reamer, further enlarging the disc space. A distractor was placed between the pedicle screws on the concave side of the lumbosacral curve; a lordotic-shaped cage filling with autologous bone graft was impacted into the disc space; the unilateral cage serves to maintain the coronal curve correction that had been obtained. The contralateral disc space on the convexity was packed with large amount of autologous bone. After the PCO plus UCS had been performed, the remainder of the procedure within the major curve was carried out in the usual fashion. Prior to securing the rods to the pedicle screws, a compressive force was applied to the screws bilaterally; the force applied on the convex side was greater at the PCO plus UCS level. Inter-transverse bone graft fusion was then performed around the surgical level.

### Statistical analysis

Data were analyzed using the Statistical Product and Service Solutions software (version 13; SPSS, Chicago, IL). Continuous variables were measured as mean ± standard deviation, and categorical variables were expressed as frequency or percentages. An independent *t* test was used to analyze the difference of continuous variables. An χ^2^ analysis and Fisher’s exact test were used to examine the differences among categorical variables. Paired *t* test was used to analyze the difference of continuous variable between preoperative and postoperative, and between 2-year follow-up and postoperative separately. Variable with *p* values smaller than 0.05 was considered to be of significant difference.

## Result

All patients underwent the operation successfully, 3 males and 29 females, mean age at surgery was 62.2 ± 7.7 years, and BMI was 25.6 ± 2.4 kg/m^2^. The PCO plus UCS was performed at L4-5 in 22 patients and at L5-S1 in 10 patients. Lower instrumented vertebrae (LIV) at L5 in 22 patients, at S1 in 10 patients, mean instrumentation level was 7.1 ± 1.6, mean surgery time was 299 ± 43 min, and mean blood loss was 1088 ± 295 mL. All the patients got back pain and leg pain alleviation and neurological function improvement at two-year follow-up (Table 1).

**Table 1:** General information of the patients

No. of cases	32
Age at surgery (years)	62.2 ± 7.7
Gender (male/female)	3/29
BMI (kg/m^2^)	25.6 ± 2.4
Coronal imbalance types	
Type A	16
Type B	9
Type C	7
PCO plus UCS	
L4-5	22
L5-S1	10
LIV (L5/S1)	22/10
No. of instrumentation levels	7.1 ± 1.6
Surgery time (min)	299 ± 43
Estimated blood loss (mL)	1088 ± 295
VAS-back	
Preoperative	5.9 ± 2.1
Two-year follow-up	1.4 ± 0.7
Paired *t* test	*t* = 12.984, *p* < 0.001
VAS-leg	
Preoperative	4.7 ± 2.8
Two-year follow-up	1.2 ± 0.6
Paired *t* test	*t* = 6.785, *p* < 0.001
ODI	
Preoperative	28.1 ± 9.1
Two-year follow-up	6.4 ± 1.7
Paired *t* test	*t* = 11.555, *p* < 0.001

Mean values are presented as ± standard deviation

*BMI* body mass index, *PCO* posterior column osteotomy, *UCS* unilateral cage strutting, *LIV* lower instrumented vertebrae, *VAS* visual analog scale, *ODI* Oswestry disability index

**Fig. 3:**
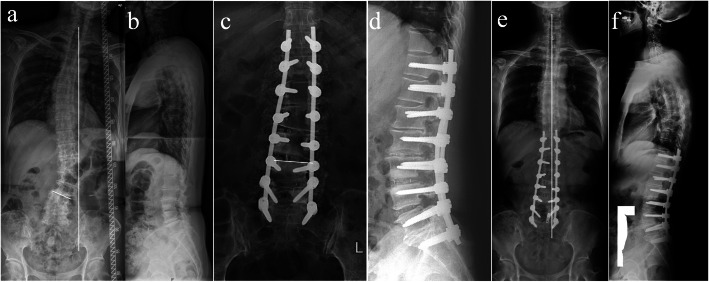
The patient, male, 63 years old. Preoperative Cobb’s angle was 31°, CBD was 47 mm, LSCA was 22°, SVA was 30 mm, LL was 26°, and LSLA was 15° (**a** and **b**). PCO plus UCS was performed at the left side of L4-5 (lumbosacral concavity). At postoperative, Cobb’s angle was 8°, LSCA was 7° (**c** and **d**). At 2-year follow-up, Cobb’s angle was 7°, CBD was 18 mm, LSCA was 7°, SVA was 14 mm, LL was 34°, and LSLA was 24° (**e** and **f**)

LSCA was changed from preoperative 20.1 ± 5.3° to postoperative 5.8 ± 5.7°, with mean correction of 14.3 ± 4.4°, and the correction was maintained at 2-year follow-up. Cobb’s angle and CBD were decreased from preoperative to postoperative; the correction was maintained at 2-year follow-up. SVA was decreased, and LL was increased from preoperative to postoperative; the correction was also maintained at 2-year follow-up. LSLA presented no change from preoperative to postoperative and from postoperative to 2-year follow-up (Table 2) (Fig. 3).

**Table 2:** Improvement of radiographic parameters from preoperative to follow-up

	CBD	Cobb	LSCA	SVA	LL	LSLA
Preoperative	22.1 ± 15.0	34.1 ± 11.4	20.1 ± 5.3	48.5 ± 50.7	− 19.9 ± 16.7	− 27.9 ± 12.6
Postoperative	16.4 ± 10.7	8.5 ± 8.2	5.8 ± 5.7	23.8 ± 26.8	− 27.1 ± 15.4	− 23.7 ± 8.3
Change (post-pre)	8.1 ± 16.1	25.6 ± 9.9	14.3 ± 4.4	25.0 ± 44.0	7.1 ± 1.3	− 4.1 ± 10.5
Paired *t* test	*t* = 2.817, *p* = 0.008	*t* = 14.500, *p* < 0.001	*t* = 18.146, *p* < 0.001	*t* = 3.212, *p* = 0.003	*t* = 2.955, *p* = 0.006	*t* = 1.132, *p* = 0.266
Two-year FU	11.8 ± 8.7	8.7 ± 8.6	5.8 ± 5.7	29.2 ± 35.0	− 24.2 ± 18.4	− 22.6 ± 8.1
Change (follow-post)	2.2 ± 8.3	0.2 ± 0.7	0.1 ± 0.4	5.7 ± 21.5	− 2.7 ± 2.9	− 1.1 ± 1.3
Paired *t* test	*t* = 1.514, *p* = 0.140	*t* = − 1.561, *p* = 0.129	*t* = − 0.373, *p* = 0.712	*t* = − 1.495, *p* = 0.145	*t* = − 0.524, *p* = 0.604	*t* = 518, *p* = 0.606

Mean values are presented as ± standard deviation

*CBD* coronal balance distance, *LSCA* lumbosacral coronal angle, *SVA* sagittal vertical axis, *LL* indicates lumbar lordosis, *LSLA* lumbosacral sagittal angle, *FU* indicates follow-up

Postoperatively, there were 8 patients with LSCA more than 10° and were enrolled into the LSCA study group; the other 24 patients were enrolled into LSCA control group. Preoperative Cobb and preoperative LSCA were larger in LSCA study group than those in LSCA control group; no difference was detected in preoperative LSCA, CBD change, Cobb change, LSCA change, Cobb change–CBD change between two sub-groups. No difference was detected in change of VAS-back, VAS-leg, and ODI between the two sub-groups (Table 3).

**Table 3:** Comparison of radiographic data between LSCA study and control groups

	LSCA study group	LSCA control group	Statistics	*p*
Preoperative CBD	24.4 ± 14.8	21.2 ± 15.3	− 0.501	0.620
Preoperative Cobb	42.4 ± 8.2	31.3 ± 11.1	− 2.590	0.015
Preoperative LSCA	27.5 ± 4.0	17.6 ± 2.9	− 7.501	0.001
CBD change	10.7 ± 21.3	7.1 ± 14.4	− 0.543	0.591
Cobb change	24.1 ± 7.3	26.1 ± 10.8	0.460	0.649
LSCA change	13.8 ± 7.3	14.4 ± 3.1	0.294	0.771
Cobb change–LSCA change	10.3 ± 3.1	11.6 ± 10.4	0.358	0.723
VAS-back change	4.7 ± 1.3	3.8 ± 1.8	− 1.265	0.216
VAS-leg change	3.1 ± 3.5	3.6 ± 2.7	0.414	0.682
ODI change	22.5 ± 15.3	17.7 ± 10.4	− 0.988	0.331

Mean values are presented as ± standard deviation

*CBD* indicates coronal balance distance, *LSCA* lumbosacral coronal angle, *VAS* visual analog scale, *ODI* Oswestry disability index

No cage subsidence was detected; all patients achieved intervertebral bone fusion and inter-transverse bone graft fusion at the lumbosacral region at final follow-up. Cerebrospinal fluid leak occurred in 1 patient (PCO plus UCS at L4-5), she received prolonged drainage, and no neurological damage was detected. Proximal junctional kyphosis (PJK) was detected in 3 patients at 2-year follow-up, they presented no symptom, and no intervention was required at that time.

## Discussion

In the current study, we prove that PCO plus UCS on the lumbosacral concavity facilitate effective correction of the fractional curve in DLS patients through complete release of dural sac as well as the asymmetrical intervertebral reconstruction by cage. The mean correction of fractional curve by PCO plus UCS was 14.3°; it was larger than the report in the previous literature (10°) [4]. According to the classification system of anatomically based spinal osteotomy by Schwab et al., the PCO procedure is classified as grade II osteotomy, which is describe as both inferior and superior facets of an articulation at a given spinal segment resected, as well as the ligamentum flavum; other posterior elements of the vertebra including the lamina, or the spinous processes, may also be resected [25]. PCO plus UCS technique not only includes the osteotomy in the posterior column of the spine, but also facilitates anterior asymmetrical intervertebral reconstruction by cage followed removal of disc; we suggest that it should be classified as grade II+ osteotomy.

There are three technical points of PCO plus UCS for lumbosacral curve correction. First, the posterior elements that include the spinous process, bilateral lamina, and the adjacent facet joints were completely removed. This degree of bone resection allows for the involved nerve roots to be completely decompressed in addition to a dorsal release of the coronal deformity and passive deformity correction [26]. When compared to the TLIF and PLIF, PCO could effectively prevent the nerve root injury on the convex side of the lumbosacral curve in the procedure of pedicle screw-rod compression. Second, using sequential reamer on the concave side, disc height could be improved, and fractional curve correction can be anticipated. Great care should be taken to preserve the vertebral bone endplates for preventing cage subsidence; this is particularly important in the setting of osteoporosis. Third, concave distraction was the preferred method of correction of the lumbosacral fractional curve over convex compression for its beneficial effects on the foraminal compression [27]. In this series, distraction of pedicle screw-rod on the concave side before anterior column support using a cage could partly correct the scoliosis; then, compression on the convex side following cage introduction could further correct the scoliosis; the cage within the concave side may act as a hinge on the coronal plane in this procedure.

Postoperative lumbosacral curve is determined by the amount of fractional curve correction and preoperative fractional curve. In the current study, all patients achieved significant fractional curve correction, but eight patients remained fractional curve more than 10° at postoperative; these patients presented larger preoperative fractional curve and major curve; the mean fractional curve correction presented no difference when compared to the other patients (13.8° vs. 14.4°). That is, the greater the coronal Cobb of the major curve, the greater the coronal Cobb of the fractional curve to compensate [3]. Although lumbosacral fractional curve can be effectively corrected by the PCO plus UCS technique, the correction is limited even though circumferential decompression plus unilateral cage strutting were performed; we suggest that it should be incorporated into the consideration of the preoperative surgical planning; complete correction of the fractional curve should not be anticipated for patients with preoperative curve more than 15°.

Both lumbosacral fractional curve correction and major curve correction in the mid-lumbar spine are critical for the postoperative coronal balance; mismatch between them may result in the aggravation of the coronal imbalance at postoperative [11, 28–30]. Wang reported that three patients had worsening of coronal balance due to straightening of the major curve without addressing the fractional curve adequately [15]. Strategies to overcome this include accepting less major curve correction or more dedicated correction of lumbosacral fractional curve [30]. Determining coronal balance intraoperatively is difficult as long cassette radiographs could not be accessed intraoperatively in many medical centers. However, an intraoperative P/A lumbar radiograph can be performed to offer the necessary visualization of lumbosacral curve correction; it could provide the surgeons with the required information to make better judgements on the overall coronal balance achieved, as the correction of major curve in mid-lumbar spine could be calculated to match for the fractional curve left at intra-operative [27]. Among the eight patients remained postoperative fractional curve more than 10° in the current study, they all achieved well postoperative coronal balance; the less major curve correction is the most likely reason.

The lumbosacral kyphotic angle slightly decreased from preoperative to postoperative followed PCO plus UCS technique; this finding is opposite to the previous reports, which proved that about 10° degrees can be corrected per level using PCO to treat a kyphotic deformity [31, 32]. Sanghyun H, et al. demonstrated that surgical level at L4-5, performing TLIF with PCO, and the preoperative kyphotic disc space angle predisposed the patients to achieve angular change more than 10° degrees sagittally [33]. The most possible explanation may be that, posterior distraction of the lumbosacral concavity through pedicle screw-rod provides direct disc height restoration and scoliosis correction, but may decrease the lordosis inevitably, since it is a kyphosing technique. By using a lordotic rod, the negative impact of the compression on the concave side would be minimized [26].

Although unilateral cage strutting could provide help in correcting the lumbosacral curve, the cage within the concave side might carry much more axial gravity loading than the autologous morserized bone being impacted within the convex side, presenting higher risk of postoperative cage subsidence on the concavity, especially in patients with osteoporosis. In the current study, no patient experienced cage migration at two2-year follow- up based on the criteria of 2 mm of cage settlement into the vertebral body, which is contrary to the above assumption. Two possible explanations may account for it. Firstly, as the interface between implants and vertebral bodies, the endplates play an important role in distributing the compressive load on the vertebral bodies. The endplate with sclerotic changes is more common on the concave side than that on the convex side of the same vertebrae, indicating that the concave side of the vertebrae may present high mechanical impedance to prevent cage subsidence [34]. Secondly, all patients achieved intervertebral bone fusion at the lumbosacral region; they also got inter-transverse bone graft fusion, which may provide additional stability at the lumbosacral region and decrease the risk of cage subsidence.

There are several potential limitations in this study. Retrospective studies suffer from a number of limitations like selection bias, and the sample size was small and from a single center, multicenter study is required to verify the surgical outcome of PCO plus UCS in the treatment of lumbosacral fractional curve in the future.

## Conclusion

PCO plus UCS on the lumbosacral concavity facilitates effective correction of the fractional curve in DLS patients through complete release of dural sac as well as the asymmetrical intervertebral reconstruction by cage.

## Data Availability

The data used and analyzed during the current study was available from the corresponding author on reasonable request.
